# Identification of phenazine analogue as a novel scaffold for thioredoxin reductase I inhibitors against Hep G2 cancer cell lines

**DOI:** 10.1080/14756366.2019.1624541

**Published:** 2019-06-09

**Authors:** Jianming Liao, Linlin Wang, Zhongxi Wu, Zhixiang Wang, Jun Chen, Yucheng Zhong, Feng Jiang, Yuanyuan Lu

**Affiliations:** a School of Life Science and Technology, China Pharmaceutical University, Nanjing, China;; b School of Engineering, China Pharmaceutical University, Nanjing, China

**Keywords:** Anticancer target, Hep G2, inhibitors, phenazines, thioredoxin reductase I

## Abstract

Even though phenazines have been extensively reported as anticancer molecules, the molecular target of these compounds is severely lagging behind. Our study consequently focuses on the anticancer target of a phenazine analogue (**CPUL1**) for its potently antitumor activities in initial stage. Along with redox status courses of Hep G2 cells, thioredoxin reductase I (TrxR1) was speculated as anticancer target of **CPUL1**. By virtue of zymologic, immunological and molecular biological experiments, we demonstrated that TrxR1 could be the anticancer target of **CPUL1**. The knowledge on phenazine targeting to TrxR1 have not been reported previously. Thus, it can provide valuable information for further development of the TrxR1 inhibitors.

## Introduction

Thioredoxin reductases (TrxR, EC 1.8.1.9) are dimeric flavoproteins belonging to the family of pyridine nucleotide-disulphide oxidoreductases, which is found in different types of cancer cells cytoplasm. TrxR overexpression is essential to maintain the phenotypes of cancer cells, and emerging evidence has demonstrated the physiological and pathological significance of TrxR1 in cellular redox signalling networks, which are involved in virtually all aspects of cell functions, such as differentiation, proliferation and death^1^. As a key member of the system, TrxR1 is the only known reductase to keep Trx1 in a reduced state, which is required for most functions of Trx1. Thus, the discovery of TrxR1 small molecular inhibitors is of importance leads to potential therapeutic agents that interfere with the cellular redox network^2^^,^^3^.

Phenazines, a large group of natural or synthetic nitrogen-containing heterocyclic compounds^4^^,^^5^, have been associated with anticancer activities since 1959^6^. Previous work suggested that the anticancer modes of the existing analogues were reported as dual topoisomerase I/II^7^ and GSH depletion^8^, respectively. Besides those targets, the knowledge on modes of action and molecular targets of phenazine analogues are severely lagging behind^9^^,^^10^.

**Figure 1.**
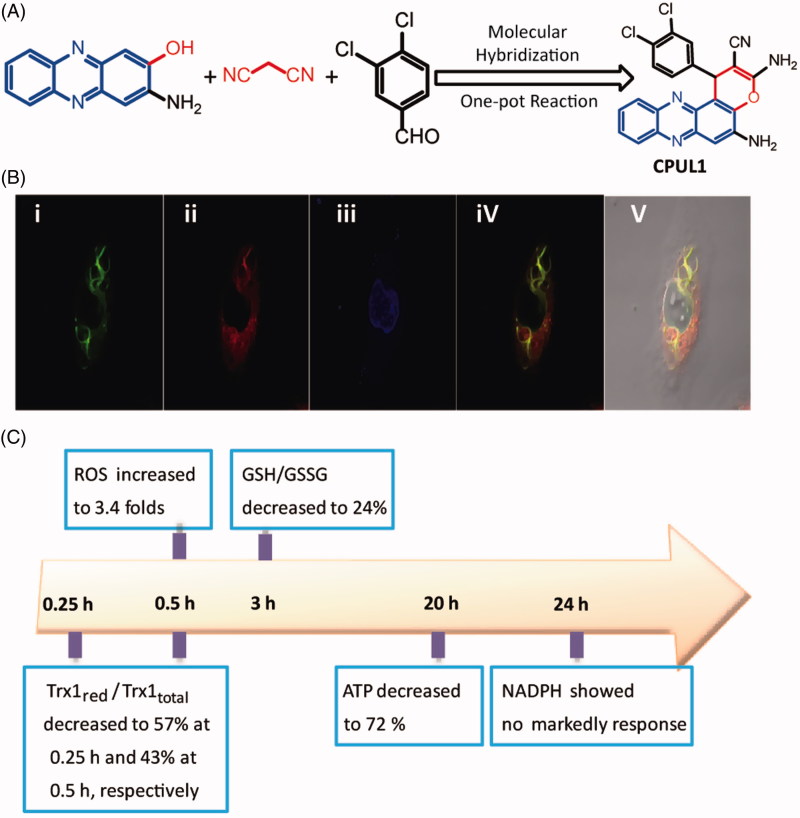
The preliminary experiment, including design strategy, LSCM and time course of the redox related key factor for investigating the target of **CPUL1**. (A) Design of ROS inducer molecule **CPUL1** with molecular hybridization strategy. (B) The distribution of **CPUL1** in the Hep G2 cells. Hep G2 cells were stained with 2 μM **CPUL1**, 0.1 μM Mito Tracker Red CMXROS, and 0.1 μ Dihydrochloride (DAPI) for 30 min. (i) Ex = 488 nm for **CPUL1**. (ii) Ex = 580 nm for Mito Tracker Red CMXROS. (iii) Ex = 360 nm for DAPI. (iv) Merged images of (i) and (iii) in dark field. (v) Merged images of (i) and (iii) in bright field. (C) A summary plot displays the time relationships between the Trx1_red_/Trx1_total_ ratio, ROS levels, GSH/GSSG ratio, NADPH lifetimes and ATP contents in Hep G2 cells treated with 2 μM of **CPUL1**.

In our previous study, a preliminary mechanism research indicated that a phenazine analogue (Figure 1(A)), namely **CPUL1**, which demonstrated antitumor activities against both mouse liver carcinoma cell lines (H22–H8D8) implanted xenografted mice *in vivo* and human liver carcinoma cell line (Hep G2) *in vitro*, might be acting as dual topoisomerase I and II inhibitors and apoptosis activator^11^. Intriguingly, with our ongoing study on the detailed antitumor molecular mechanism of compound **CPUL1**, we found that the compound was prevailingly distributed thoroughly in Hep G2 cell plasma not in cytoplast (Figure 1(B)), which was confirmed by laser scanning confocal microscopy (LSCM). This freakishly phenomenon was distinguishing from typical topoisomerase I/II inhibitors, such as doxorubicin^12^, etoposide^13^ and 10-hydroxycamptothecin^14^, which were reported as locating at nucleus in cancer cell lines by LSCM methods. The discrepant results of **CPUL1** between the LSCM and topoisomerase I/II inhibition experiments aroused a suspicion that the **CPUL1** might not targeting to the topoisomerase I/II in Hep G2 cell lines. Considering the controversial role of the **CPUL1** against Hep G2 cells, the target of **CPUL1** against Hep G2 cells becomes the crux of the scene to be unveiled. Thus, we attempted to discover and identify the anticancer target of **CPUL1** in this study.

## Materials and methods

The general procedures, the details concerning the experiment steps and the analytical data are provided in the Supplementary Material.

## Results and discussion

Since we observed visible apoptosis of Hep G2 cells after treated with **CPUL1**, we sought to find clues from the process of redox status. We tested the time courses of redox related key factors in Hep G2 cells, among them ROS levels, GSH/GSSG ratios, NAPDH levels and ATP levels before and after treated with **CPUL1** at different time, respectively (Figure 1(C) and Figures S1–S4, see Supplementary Material). In these results, most unexpectedly, the ROS levels were dramatically increased at the first 15 min (Listed in Figure 1(C) and Figure S1). However, NADPH (Figure S4) and ATP levels (Figure S2) did not show significant differences with control groups before 18 h, respectively. It is widely recognized that the depletion of NADPH and ATP is associated with the pace of apoptosis^15^^,^^16^. However, the stable NADPH and ATP levels in the first 4 h after treated with **CPUL1** can deduce a result that ATP mediating the ROS produce process did rather not take place in HepG2 cells after treated by **CPUL1**. Combined the results of the redox related key factors time-course study, a conjectural apoptosis process was hypothesized as following: (1) **CPUL1** could trigger apoptosis mainly through elevating the ROS level rather than inhibiting the topoisomerase I/II; and (2) deleting ROS function instead of accelerating ROS production might be inhibited by **CPUL1** in apoptosis cells.

**Figure 2.**
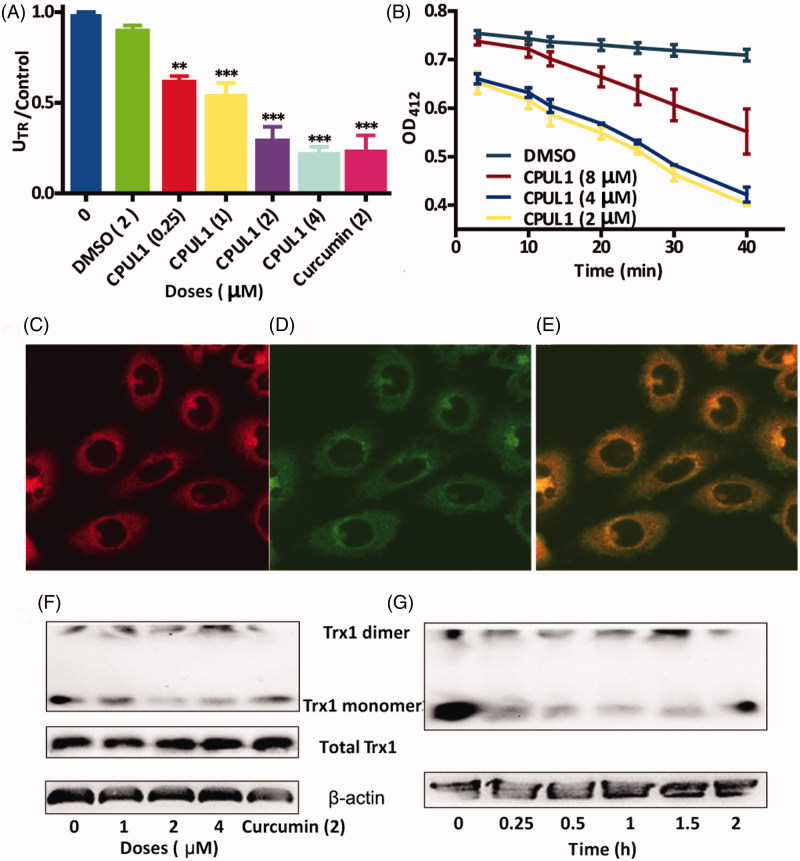
The evidences for **CPUL1** acted as TrxR1 inhibitors based on enzymatic reaction, immunofluorescence and non-reduced western blot. **(**A) TrxR1 activities vs control after treated with different doses of **CPUL1** (0.25, 1, 2 and 4 μM) for 0.5 h. (B) The time course NADPH consumption after treat with different doses of **CPUL1** (2, 4 and 8 μM) for (10, 20, 30 and 40 min), respectively. (C–E) The immune-fluorescence staining of Hep G2 after treated with TrxR1 antibody (C, 580 nm), **CPUL1** (D, 448 nm) and merged images (E), respectively. (F, G) The non-reduced western blot assays of Trx1, including the Hep G2 cells were treated with **CPUL1** at different doses (1, 2 and 4 μM) for 0.5 h (F) and treated with 4 μM **CPUL1** for different time (0, 0.25, 0.5, 1, 1.5 and 2 h) (G), respectively. Curcumin used as positive control. Values represent the mean** **±** **SD obtained from three different experiments. *p < 0.05, **p < 0.01, ***p < 0.001 significantly different from the value of control (untreated).

In mammalian cells, there are two major thiol-dependent antioxidant systems, the thioredoxin- (Trx) and the glutathione- (GSH) dependent enzyme systems which may act in concert^17^^,^^18^. In the next experiment, we tried to verify if there were significant differences between Trx1_red_/Trx1_total_ and GSH/GSSG levels under treatment of **CPUL1** in Hep G2 cell lines. Amazingly, Trx1_red_/Trx1_total_ levels decreased to 57% at 0.25 h and 43% at 0.5 h (Figure 2(G)), whereas, GSH/GSSG ratios are markedly decreasing after 2 h (Figure S3), respectively. These results can be elucidated that the reductive Trx1 level decreased dramatically at the first 0.5 h, and the ROS level simultaneously increased by 3.4-folds, then the GSH compensation mechanism had come into force and decreased to 24% after 2 h. Harris^18^ and Mandal^19^ have also demonstrated homoplastically standpoint that the Trx1 and GSH can work synergistically as antioxidant roles, as long as the GSH metabolism could compensate the lack of reductive Trx1 in tumour cells.

**Figure 3.**
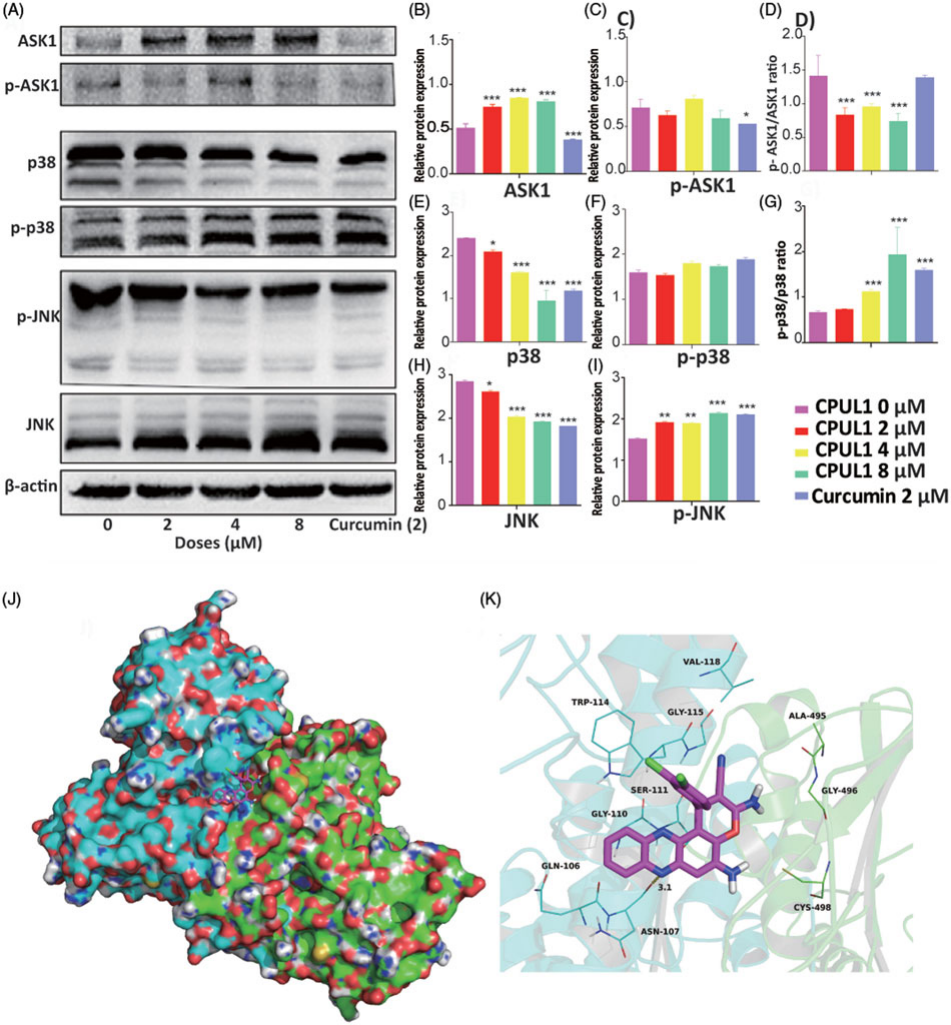
The results of Western blotting key ROS stress proteins and **CPUL1** docked into the binding site of the TrxR1. (A) Western blotting results of ASK1, p-ASK1, p38, p-p38, p-JNK and JNK after treated by different doses of **CPUL1** (2, 4 and 8 μM). Curcumin used as positive control. (B–I) The levels of proteins in (A) were normalized to β-actin. Values represent the mean** **±SD obtained from three different experiments. *p < 0.05, **p < 0.01, ***p < 0.001 significantly different from the value of control (untreated). (J) Total view of CPUL1 was docked into the binding site of the TrxR1. (K) Detailed view of CPUL1 was docked into the binding site of the TrxR1.

In the following section, we focused on the crucial proteins in Trx enzyme system including TrxR1 and Trx1, which are widely scattered in cytoplasm of cells. We tried to detect the mRNA levels of TrxR1 and Trx1 treated with **CPUL1** by RT-PCR, in order to see whether the gene expression of both proteins were disturbed by **CPUL1** at the very beginning. The results showed that the mRNA levels of the two groups were not significantly different from those of the negative control group (the data were not shown).

The existing TrxR1 inhibitors^18^^,^^20–22^ had inspired us to detect the TrxR1 activities, including free-cell and in-cell experiments, after treated with **CPUL1**, respectively. Surprisingly, the **CPUL1** could inhibit the TrxR1 activities in both dose- and time-dependent manners against negative and positive groups (Figure 2(A,B)), respectively. In free-cell experiment (Figure 2(A)), the TrxR1 activities was decreased to 19.5% against control groups after treated with 4 μM **CPUL1**. In the time-course experiment, we chosen the in-cell experiment to detect the variation of TrxR1 activities along with time of **CPUL1** treament, in which the consumption of NADPH was in direct proportion to the activities of TrxR1^23^. Figure 2(B) demonstrated time course of NADPH consumption (OD_412 nm_) in the biomedical reaction system, in which we could see that the OD_412 nm_ of NADPH was dramatically decreased along with the doses of **CPUL1** decreased from 2 to 8 μM; it simultaneously means that the TrxR1 reductive activites was decreased along with the increased doses of **CPUL1**.

Secondly, the immunofluorescence staining experiment was performed to prove the combination of **CPUL1** and TrxR1, in which the TrxR1 was traced by its fluorescent antibody by incubated with Hep G2 cells for 2 h. Figure 2(C,D) indicated the fluorecence of TrxR1 antibody and **CPUL1** in the Hep G2 cells at absorption of 580 and 448 nm, respectively. When combining Figure 2(C,D) together in Figure 2(E), we can see that the fluorescence of TrxR1 antibody was perfectly merged with **CPUL1**, which adamantly suported the evidence of that **CPUL1** could bind to TrxR1 in the cells.

Lastly, Trx1, the crucial downstream protein of TrxR1, was further investigated to evidence the TrxR1 was inhibited by the compound. To the best of our knowledge, the oxidative status of Trx1, the downstream protein of TrxR1, could be demonstrated by non-reducing westernblot^24^. As we can see from Figure 2(F,G), the total Trx1 protein expression showed no significant difference between the treated groups and untreated groups, so total Trx1 of the HepG2 cells were not affected by treatment of the **CPUL1**; however, the monomer of Trx1 ratio was decreased and Trx1 dimer ratio was increased after treated with **CPUL1** in both dose- and time-dependent fashion against negative and positive control groups (Figure 2(F,G)), respectively. The non-reduced western blot straitforwardly demonstrated that the oxidative status of Trx1 was increased after treated with **CPUL1**. These results indicated that redox homeostasis of the Hep G2 cells was broken to trigger the apoptosis of the cancer cells.

The TrxR1 activties might be inhibited at the very beginning, then the preponderance of Trx1, the crucial downstream protein of TrxR1, was following transfer to oxidative status, which disequilibrated redox status of the Hep G2 cells. In order to figure out the anti-proliferation funtion of the **CPUL1** in the HepG2 cells, the downstream protein molecules of the Trx1 were further investigated in the following western blot experiments (Figure 3(A)).

At the begining of the protein profiles of the downstream protein molecules, ASK1, a member of mitogen-activated protein kinase (MAPK) family, was chosen as the first molecule to be investigated, which is not only combined to reduce Trx1 directly but also can induce apoptosis in cancer cells at free molecule status^25^. Since reduced Trx1, but not the oxided Trx1 is known to bind with ASK-1 to repress its activities^26^, we firstly obtained the potein levels of ASK-1 before and after treated with **CPUL1** by western blot analysis. In agreement with this, increased levels (1.6 folds) of ASK1 were found in HepG2 cells after exposed to 4 µM of **CPUL1** for 24 h (Figure 3(A,B)), which is consistent with the increased oxidation levels of Trx1 previously observed in Figure 2(D).

Next, we tried to detect the phosphorylation levels of ASK1 which could indicate ASK1 activities in the cells, wheras the phosphorylation levels of ASK1 did not show significant difference versus negative control groups (Figure 3(A,C)). However, to our surprise, the pASK1/ASK1 ratio decreased to 68% after incubated 4 µM of **CPUL1** after 24 h compared to negative control groups (Figure 3(D)), implying that the activities of ASK1 were engaged after exposed to **CPUL1**.

Then, JNK and p38, the downstream proteins of ASK1 in mammalian cells^27^, were selected to gain further insight into the molecular mechanism of anti-tumor activity of **CPUL1** in the following western blotting experiments, respectively. Our results showed that 4 µM of **CPUL1** activated the phosphorylation of JNK (1.3-folds vs. blank control) and decreased JNK levels to 29% in concentration-dependent manner (Figure 3(A,H and I)), respectively. The expression levels of p38 were decreased to 67% comparing to blank control groups (Figure 3(E)), wheras the phosphorylation of p38 (Figure 3(F)) did not show significant differences between the experiment groups and blank control groups. However, the ratio of p-p38/p38 were increased singnificantly along with the doses of **CPUL1** rising (Figure 3(G)). These results suggested that the ROS levels fortified by the **CPUL1** appeared to enhance JNK and p38 signals to sensitize Hep G2 cell apoptosis.

We and others^28^ have implicated JNK and p38 MAPK pathway in the apoptotic effects in many human cancer cells, which were similarly caused by TrxR inhibition. Besides that, Al-Gayyar^29^ demonstrated that up-regulation of thioredoxin interacting protein (TXNIP), an endogenous inhibitor of Trx, could decrease Trx1 activities and activate the pro-apoptotic p38 MAPK/JNK pathway. Combined above results of the protein profiles, these effects implied that the anticancer mechanism phenomena of **CPUL1** is identical to those existing TrxR1 inhibitors^26^^,^^28^.

From above evidences, we preliminarily verified the **CPUL1** could combine with TrxR1 and inhibit it at enzymes levels, cell levels and molecular levels, respectively. As far as we known, there are three main classes of TrxR1 inhibitors according to past research results^30^. The first class of TrxR1 inhibitors is binding to Sec-498 in TrxR1, such as Auranofin^18^ and iron(II) complexes^22^. The second type of TrxR inhibitors, for instance Curcumin^21^ and Brevetoxin 2^20^, can attack both Sec-498 and Cys-58 active sites of TrxR. 4-hydroxy-2-nonenal^31^, quinols^32^ and Se compounds^33^, the third genre of TrxR inhibitors, could inhibit TrxR as well as Trx without selectivities.

In order to investigate the binding mode between **CPUL1** and TrxR1, the theoretical binding mode of **CPUL1** in the binding site of the TrxR1 was illustrated in Figure 3(J,K). **CPUL1** adopted a compact conformation to bind in the site of the TrxR1. **CPUL1** was located at the hydrophobic pocket, surrounded by the residues Trp-114, Val-118, Ala-495 and Sec-498, forming a stable hydrophobic binding. Detailed analysis showed that the 3,4-dichlorophenyl group of **CPUL1** formed CH–π interaction with the residue Trp-114. One of the chlorine atom of **CPUL1** formed Cl–π interaction with the residue Trp-114. Importantly, one hydrogen bond was observed between the **CPUL1** and residue Asn-107, with bond lengths of 3.1 Å, which was the main interactions between the **CPUL1** and the TrxR1. To our surprise, the key double bond was oriented to the residue Sec-498, which was convenient for the selenocysteine to be attacked. All these interactions helped **CPUL1** to anchor in the binding site of the TrxR1. The above molecular simulations gave us rational explanation of the interactions between **CPUL1** and TrxR1, which provided valuable information for further development of the TrxR1 inhibitors.

All together, with the time course of the redox related key factor strategy, we disclosed phenazine analogue **CPUL1** might target to the TrxR1, which was further evidenced by zymological, immunological and molecular biological experiments. To the best of our knowledge, phenazine analogues have not been reported as TrxR1 inhibitor in previous study. Lastly, we tried to cope with the characteristic of the combination between **CPUL1** and TrxR1, and the results showed that **CPUL1** was formed a stable hydrophobic domain between the residues Trp-114, Val-118, Ala-495 and Sec-498, which is valuable for further development of the TrxR1 inhibitors.

## Supplementary Material

Supplemental Material
