# Supplementary material for: Identification of phenazine analogue as a novel scaffold for thioredoxin reductase I inhibitors against Hep G2 cancer cell lines
Source: J Enzyme Inhib Med Chem. 2019 Jun 9;34(1):1158–63. doi: 10.1080/14756366.2019.1624541 (PMC6567043; doi:10.1080/14756366.2019.1624541)
Supplement: Supplemental Material [file IENZ_A_1624541_SM2183.pdf]

Electronic Supplementary Information

for

Identification of phenazine analogue as a novel scaffold for thioredoxin reductase I inhibitors against Hep G2 cancer cell lines

Jianming Liao,[a†] Linlin Wang,[a†] Zhongxi Wu,[b] Zhixiang Wang,[b] Jun Chen,[a]

Yucheng Zhong,[a] Feng Jiang*[b] and Yuanyuan Lu*[a]

a. School of Life Science and Technology. China Pharmaceutical University, Nanjing 210009, China.
b. School of Engineering. China Pharmaceutical University, Nanjing 210009, China.

1. Experiment sections

1.1 Cell culture and reagents

HepG2 cells were purchased from the Cell Bank of the Shanghai Institute of Cell Biology. HepG2 cells were cultured in DMEM medium (Gibco/BRL, Grand Island, New York, USA) supplemented with 10% fetal bovine serum (FBS, Gibco/BRL, v/v), penicillin (100 units/mL), and streptomycin (100 mg/L). Cells were maintained at 37 °C in a humidified atmosphere of 5% CO_2. Compounds were dissolved in DMSO as a 100 mmol/L stock solution was used for the treatment of cells. In solvent control group, cells were treated with 0.1% DMSO.

1.2 Laser-scanning confocal microscopy

HepG2 cells were separately cultured by using the same medium at 37 °C 20% CO_2. They were cultured 2 days. Then, the living HepG2 cells were incubated in a confocal dishes, with either of the compound 1 (2, 4, and 8 μM), Mito Tracker Red CMXROS (0.1 μM, Yeasen, Shanghai, China) in culture medium for 30 min, respectively. Finally, these cells were used for cell imaging using confocal microscopy.

1.3 Time course of ROS detection

HepG2 cells were cultured 0 h, 0.5 h, 1 h, 2 h, 3 h, 4 h, 5 h, 6 h and 7.5 h in 6-well plates, after added 0.25 μM CPUL1. After centrifuged the suspended cells, washed the adherent cells with PBS twice, trypsin digested the cells, collecting all the cells at 4 °C, 10,000 g, 5 min. All the samples were added into 96- wells black ELISA plates. After incubating with MitoSOX probe 15 min without light, all the sample's fluoresce strength was detected by Infinite 200 PRO (TECAN, Switzerland), Ex/Em= 510/580 nm. ROS strength levels are expressed as (Δ_A-$\Delta_{control}$)/μg protein, and the data are presented as the average of three independent experiments.

1.4 Time course of ATP detection

The ATP levels detection was performed by ATP assays kit (Beyotime, China, Cat No. S0026), according to the manufacturer's protocol. ATP content is expressed as nmol/mg protein, and the data are presented as the average of three independent experiments.

1.5 GSH/GSSG ratio detection

Reduced (GSH) and oxidized (GSSG) glutathione levels in HepG2 cells were measured through colorimetric determination by using GSH and GSSG Assay Kit (Beyotime) [1, 2]. Briefly, 40 μL metaphosphoric acid was added into 10 μL the suspended cells and then centrifuged at 10,000 g for 10 min at 4 C. The supernatant was used for GSH and GSSG assay. The total GSH level was measured by the method of DTNB-GSSG recycling assay (Baker et al. 1990). The GSSG level was quantified by the same method of total GSH assay after the supernatant was pretreated with 1% 1 mol/L 2-vinylpyridine solution to remove the reduced GSH. The amount of reduced GSH was obtained by subtracting the amount of GSSG from that of the total GSH.

1.6 NADPH detection

The NADPH was measured by a commercial NADPH quantification kit (#MAK038; Sigma-Aldrich). Briefly, the cells were extracted with extraction buffer, homogenized, and centrifuged at 10,000 g for 10 min to isolate the NADPH/$NADP^+$-containing supernatant. An aliquot of the supernatant was heated at 60 °C for 30 min to decompose the $NADP^+$, cooled on ice, and spun quickly to remove the precipitate. Another aliquot of the supernatant was not heated. Both aliquots were reacted with $NADP^+$ cycling buffer and enzyme mix (containing glucose-6-phosphate dehydrogenase G6PDH]) for 5 min at room temperature to convert $NADP^+$ to NADPH. The solutions were then incubated with NADPH developer for 2 h and the absorbance measured at 450 nm. The amount of NADPH (heated sample) and the total $NADP^+$ and NADPH (unheated sample) were quantified from an NADPH standard curve. The data are presented as the average of three independent experiments.

1.7 qRT-PCR

RNAs were extracted from GMCs using Trizol (TAKARA, Japan). 1 µg RNA was reversed transcripted using Bestar qPCR RT Kit (DBI, USA) on ABI 9700 PCR (DBI, USA). Real time PCR was performed using EzOmics® Sybr qPCR master Mix Kit (EZBioscience, USA) at 20 µL system on Real time PCR system (Stratagene Mx3000 P, Agilent, USA). Following primer was used in this study: GDAPH, 5′-AAGGTCGGAGTAACCGGATT-3′ forward, 5′-CTGGAAGATGGTGATGGGATT-3′ reversed; Trx1, 5′-AGTAGACGTGGATGACTGCC-3′ forward, 5′−CCAGAGAACTCCCCAACCTT-3′ reversed; TrxR1, 5′-TTGAAGCAGGGACACCA-3′ forward, 5′-GCCAGCATCACCGTATT-3′ reversed. The PCR was performed at 95°C 5 min, 95°C 30 s; 30 circles of 72°C 10 min. Each sample was detected in triple and expression was calculated using $2^{-\Delta\Delta Ct}$ and normalized to control.

1.8 Free-cell TrxR1 activities

The TrxR1 activities were detected according to TrxR1 kit assay (IMCO, Sweden). Briefly, added Trx and TE buffer (50 mM Tris-Cl and 20 mM EDTA) or protein buffer in each well, and added 20 µL of reaction mixture (20 µL 40 mg/mL β-NADPH mixed with 480 µL mixture M (210 mM HEPES PH 7.6, 790 µM Insulin (4.5 mg/mL) and 20 mM EDTA) (Beyotime, China), then incubated the samples with **CPUL1** and Curcumin immediately for 30 min at 37 °C. At the end of the incubation added 200 µL stop solutions (180 µL 8 M GuHCl in 0.2 M Tris-Cl (pH 8.0) and 20 µL 10 mM DTNB in 99.5% ethanol) to each well. Read the absorbance at 412 nm of the standard curve and the samples using micro plate reader.

1.9 TrxR1-inhibition activities in cell lysate

The TrxR1-inhibitory activities in cell lysate were detected using an endopiont insulin reduction assay [3, 4]. HepG2 cells were incubated with different concentrations (2, 4 and 8 µM) of **CPUL1** for 6 h in the incubator. The control group contained the same amount of Me_2SO (1%, v/v). Before harvesting, cells were washed with phosphate-buffered saline and then cells were lysed with cell

lysis buffer (0.5% Triton X-100, 0.5% deoxycholate, 0.1% SDS, 150 mM NaCl, and 1 mM EDTA in TE buffer) in the presence of protease inhibitors (Complete, EDTA-free, catalog number 1873580, Roche Applied Science). The activity of TrxR1 in the cell extracts was determined as described elsewhere [3, 4]. Protein concentration was quantified using the Bio-Rad procedure. A volume corresponding to 50 µg of protein from each cell extract was incubated with TE buffer containing 300 µM NADPH, 1.5 mg/ml insulin, and 17 µM Escherichia coli Trx1 at room temperature for 30 min in a final volume of 40 µl. By the addition of 500 µl of 1 mM DTNB in 6 M guanidine hydrochloride (pH 8.0) the reaction was terminated. A blank sample, containing everything except Trx1, was treated in the same manner. The absorbance at 412 nm was measured, and the blank value was subtracted from the corresponding absorbance value of the sample. The activity of the enzyme was expressed as U_{TR}/the control, and the data are presented as the average of three independent experiments.

1.10 Immunofluorescence of TrxR1 in HepG2 cells

Cells in 24 - hole chambers were fixed in 4% paraformaldehyde for 10 min and blocked with 0.2% Triton X-100 dissolved in 1% BSA for 30 min. Then, cells were incubated with primary antibodies overnight at 4°C. After rinsing several times with PBS, cells were incubated with Rhogamin-labeled goat anti-rabbit IgG (red) for 2 hr. Images were obtained with a Leica TCS SPE II fluorescence microscope (Leica, Germany). Immunofluorescence was performed using the following primary antibodies: Rabbit monoclonal antibodies, anti-TrxR1 was purchased from Proteintech (United States).

1.11 None reduced western blotting of Trx1

Following treatment, HepG2 cells were collected, resuspended in 75 µL RIPA+PMSF (100:1) lysis buffer and then incubated for 30 min and every 5 min vortex to well distributing. Insoluble material was removed by centrifugation at 12,000 × rpm for 15 min. Protein extracts (65 µL) were combined with non-reducing sample loading buffer (62.5 mM Tris-HCl pH 6.8, 10% glycerol, 2% SDS, 0.025% bromophenol blue) and resolved by SDS-PAGE (15% gel). Proteins were transferred to PVDF membrane which was subsequently blocked with 5% skim milk in Tris-buffered saline containing 0.05% Tween20 (TBST). Blots were probed with the appropriate Trx1 antibody in TBST containing 5% BSA and immunoblotted proteins were visualized using a rabbit-Trx1-conjugated secondary antibody and the ECL™ system. Images were obtained using a ChemiDoc™ XRS system and Quantity One® software (Bio-Rad Laboratories, Hercules, CA) was utilized to quantify the oxidation state of the Trx1.

1.12 Western blotting of key proteins

HepG2 cells (3×10^5 cells/dish) treated with **CPUL1** were lysed and collected using RIPA lysis buffer. BCA protein assay kit was used to determine concentrations of protein samples. Proteins were resolved by 12% SDS-PAGE and electroblotted onto nitrocellulose membranes. The blots

were blocked with 5% non-fat dry milk for 2 h at room temperature and then incubated overnight with specific antibodies in TBST overnight at 4 °C. After washing, chemiluminescent detection was recorded using ECL kit (Bio-Rad, Hercules, CA). Primary rabbit monoclonal antibodies of β-actin, ASK-1, p-ASK1, p38, p-p38, JNK and p-JNK were purchased from Wanlei Biotech Inc (China).

1.13 Molecule docking of the TrxR1 and **CPUL1**

Molecular docking studies were performed to investigate the binding mode between the **CPUL1** and human thioredoxin reductase I (TrxR1) using Autodock vina 1.1.2[5]. The three-dimensional (3D) coordinate of the TrxR1 (PDB ID: 2ZZB) was downloaded from Protein Data Bank (http://www.rcsb.org/pdb/home/home.do). The 3D structure of **CPUL1** was drawn by ChemBioDraw Ultra 12.0 and ChemBio3D Ultra 12.0 software. The AutoDockTools 1.5.6 package was employed to generate the docking input files[6,7]. The search grid of the TrxR1 was identified as center-x: -20.734, center-y: 0.046, and center-z: 0.028 with dimensions size-x: 15, size-y: 15, and size-z: 15. The value of exhaustiveness was set to 20. For Vina docking, the default parameters were used if it was not mentioned. The best-scoring pose as judged by the Vina docking score was chosen and visually analyzed using PyMoL 1.7.6 software (http://www.pymol.org/).

2. Results

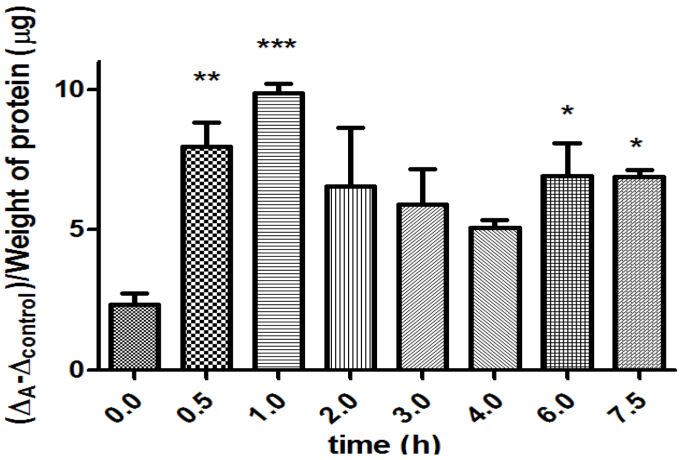

Fig. S1 The time course of ROS levels in HepG2 cells **CPUL1** 4 μM at 0, 0.5, 1.0, 2.0, 3.0, 4.0, 6.0 and 7.5 h. Values represent the mean ± SD obtained from three different experiments. *P<0.05, **P<0.01, ***P<0.001 significantly different from the value of control (untreated).

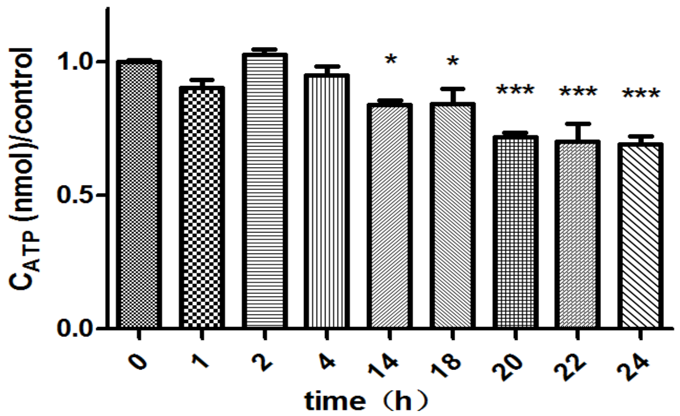

Fig. S2 The time course of ATP levels in HepG2 cells after treated with **CPUL1** 4 μM at 0, 1, 2, 4, 14, 18, 20, 22 and 24 h. Values represent the mean ± SD obtained from three different experiments. *P<0.05, **P<0.01, ***P<0.001 significantly different from the value of control (untreated).

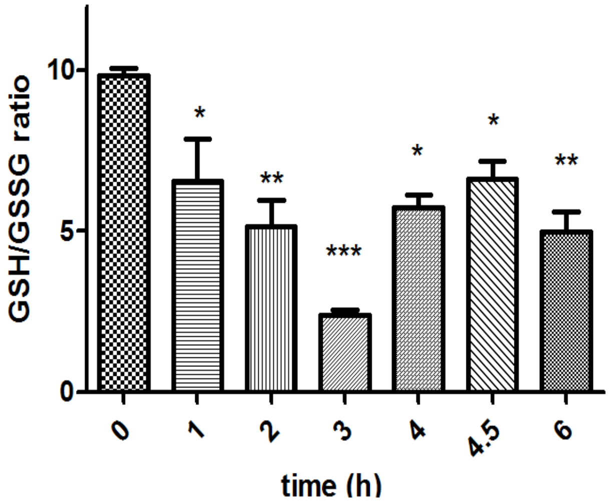

Fig. S3 The time course of GSH/GSSG ratios in HepG2 cells after treated with **CPUL1** 4 μM at 0, 1, 2, 3, 4, 4.5, 6 h. Values represent the mean ± SD obtained from three different experiments. *P<0.05, **P<0.01, ***P<0.001 significantly different from the value of control (untreated).

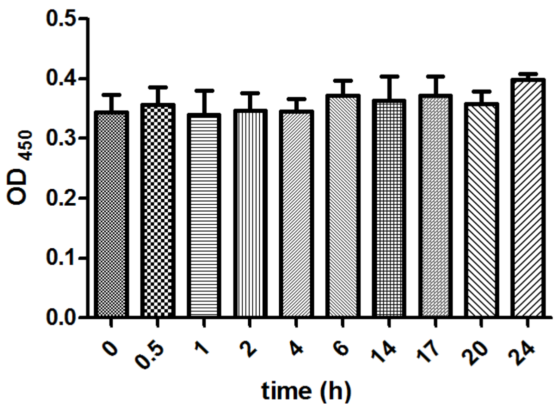

Fig. S4 The time course of NADPH levels in HepG2 cells after treated with **CPUL1** 4 μM at 0, 0.5, 1, 2, 4, 6, 14, 17, 20 and 24 h. Values represent the mean ± SD obtained from three different experiments. *P<0.05, **P<0.01, ***P<0.001 significantly different from the value of control (untreated).

Refrences

1. H. W. Jeong, K. C. Hsu, J. W. Lee, M. Ham, J. Y. Huh, H. J. Shin, W. S. Kim, J. B. Kim. Am J Physiol Endocrinol Metab 2009, 296, E955–E964.

2. B. Wang, W. Feng, M. Zhu, Y. Wang, M. Wang, Y. Gu, H. Ouyang, H. Wang, M. Li, Y. Zhao, Z. Chai, H. Wang. J Nanopart Res, 2009, 11, 41-53.

3. J. Fang, J. Lu and A. Holmgren. J Biol Chem, 2005, 280, 25284-25290.

4. W. J. Yan, Q. Wang, C. H. Yuan, F. Wang, Y. Ji, F. Dai, X. L. Jin, B. Zhou. Free Rad Biol Med, 2016, 97, 109-123.

5. O. Trott, A. J. Olson. J Comput Chem 2010, 31, 455-61.

6. M. F. Sanner. J Mol Graph Model 1999, 17, 57-61.

7. G. M. Morris, R. Huey, W. Lindstrom, M. F. Sanner, R. K. Belew, D. S. Goodsell, A. J. Olson. J Comput Chem 2009, 30, 2785-91.
